# Presence and biodistribution of perfluorooctanoic acid (PFOA) in *Paracentrotus lividus* highlight its potential application for environmental biomonitoring

**DOI:** 10.1038/s41598-021-98284-2

**Published:** 2021-09-21

**Authors:** Dario Savoca, Raffaella Melfi, Antonio Palumbo Piccionello, Salvatore Barreca, Silvestre Buscemi, Vincenzo Arizza, Marco Arculeo, Andrea Pace

**Affiliations:** 1 Dipartimento di Scienze e Tecnologie Biologiche, Chimiche e Farmaceutiche (STEBICEF), Università degli Studi di Palermo, 90100 Palermo, Italy; 2 Department of Pharmaceutical Sciences, Università degli Studi di Milano, Via L. Mangiagalli 25, 20131 Milan, Italy

**Keywords:** Ecology, Environmental sciences, Environmental chemistry, Environmental impact

## Abstract

The first determination of presence and biodistribution of PFOA in ninety specimens of sea urchin *Paracentrotus lividus* from two differently contaminated sites along Palermo’s coastline (Sicily) is reported. Analyses were performed on the sea urchins’ coelomic fluids, coelomocytes, gonads or mixed organs, as well as on seawater and *Posidonia oceanica* leaves samples from the collection sites. PFOA concentration ranged between 1 and 13 ng/L in seawater and between 0 and 794 ng/g in *P. oceanica*. The analyses carried out on individuals of *P. lividus* from the least polluted site (A) showed PFOA median values equal to 0 in all the matrices (coelomic fluid, coelomocytes and gonads). Conversely, individuals collected from the most polluted site (B) showed median PFOA concentrations of 21 ng/g in coelomic fluid, 153 ng/g in coelomocytes, and 195 ng/g in gonads. Calculated bioconcentration factors of log_10_BCF > 3.7 confirmed the very bioaccumulative nature of PFOA. Significant correlations were found between the PFOA concentration of the coelomic fluid versus the total PFOA concentration of the entire sea urchin. PERMANOVA (*p* = 0.001) end Welch's t-test (*p* < 0.001) analyses showed a difference between specimens collected from the two sites highlighting the potential application of *P. lividus* as sentinel species for PFOA biomonitoring.

## Introduction

Emerging and persistent organic pollutants (POPs) are a threat to the marine environment and strategies are needed for their periodical and sustainable monitoring^1,2^. Perfluoroalkylated substances (PFAS) are a subclass of POPs broadly distributed in the environment due to their extensive usage in a wide variety of applications and products (from food-packaging to electronics)^3^. Due to the strength and stability of the carbon–fluorine bond, PFAS are generally resistant to hydrolysis, photolysis, or microbial degradation, and are highly persistent in all environmental compartments, especially water^3,4^. PFAS have been also found in wildlife^5,6^ fish and other seafood^7–9^ and have a high affinity for sediments and organic matter due to chemical adsorption phenomena^10,11^. Additionally, PFAS can potentially interfere with normal reproductive and hormonal functions as endocrine disruptors, showing adverse effects, for example PFOA exposure is associated with kidney and testicular cancer in humans^12^.

In the majority of studies reporting PFAS contaminated matrices^8,9,13^ perfluorooctanoic acid (PFOA) is among the most frequently detected compounds at concentration levels significantly higher than other minor fluorinated pollutants. Probably this situation will continue to be recorded despite PFOA (its salts and related-compounds) was added to Annex A of the Stockholm Convention in 2019 and its use has recently been limited by the European legislation^14^. In this context the monitoring of PFOA in the environment could provide a more reliable evaluation of fluorinated pollutant contamination since PFOA can also result from the degradation of other fluorinated compounds^15^. Indeed, the widespread occurrence of PFOA-related compounds represents a potential threat for the environment especially to aquatic ecosystems organisms.

Indeed, PFOA is easily absorbed by aquatic organisms, and its elimination depends on physiological mechanisms that are different among species and sexes^16,17^. Several marine organisms were investigated for PFOA levels^10,18,19^, although non-invasive sampling is not always possible with all the species and not all the species were indicative of the pollution status of the collection area. In fact, the mobility characteristics of an animal, its sedentarity and longevity are crucial in the approach to biomonitoring studies since an ideal living bioindicator should be strongly connected to the local environment and survive enough time to allow for multiple periodical sampling^20,21^.

Moreover, PFOA is present in the sea, with an average concentration from a few units to hundreds of ng/L with higher values in river basins and in the coastal area of industrial cities^22,23^.

In the frame of our research on the presence of POPs in marine animals^24^, we focused our study on the marine *Paracentrotus lividus* (Lamarck, 1816) widely used in ecotoxicological studies due to its wide distribution, sedentary lifestyle and longevity (about 15 years)^25^. Additionally, the gonads of *P. lividus* are consumed as seafood in different Mediterranean countries thus posing, if contaminated, a potential threat to human health^26^. Moreover, coelomocytes, i.e. the cells freely circulating in the coelom of *P. lividus,* can be used as biosensors of environmental stress^27–29^.

Recently, the contamination levels of several emerging and persistent pollutants in wild *P. lividus* have been reported^30^. Conversely, studies on the biodistribution and uptake of fluorinated pollutants by adult *P. lividus* specimen from the wild marine environment have never been conducted.

The aim of this research was to investigate the presence and distribution of PFOA in *P. lividus*, *Posidonia oceanica* (Linnaeus) Delile, 1813, seawater and brackish water samples collected from the coastal area near Palermo (Sicily).

## Materials and methods

### Samples collection

**Figure 1:**
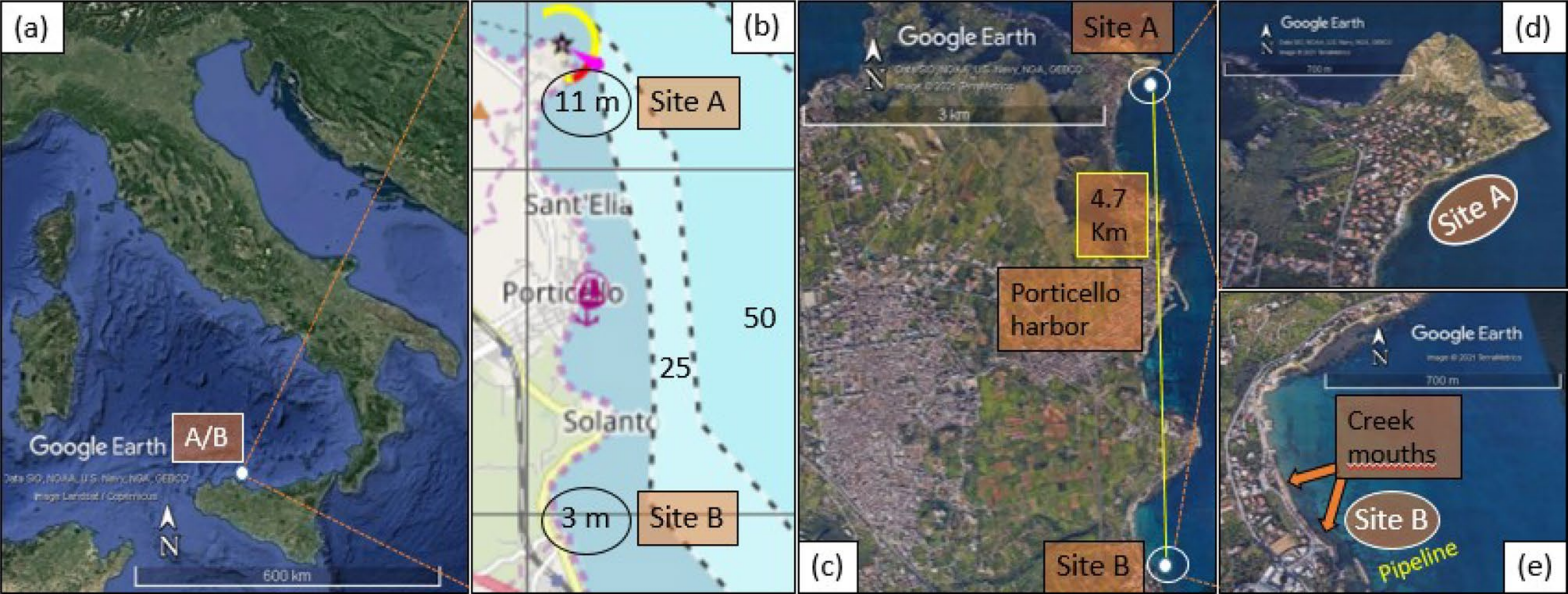
Map of the sampling site. (**a**) Geographic area, (**b**) bathymetric chart and (**c**) relative distance between sample sites; (**d**, **e**) close ups of sampling sites. (Images obtained by courtesy of Google Earth Pro and map.openseamap.org).

Three sampling campaigns were carried out at the two sample sites (A and B) on the coast of north-western Sicily (Fig. 1a) chosen for this study. The main features of the sites and sampling details are summarized in Table S1 (Supplementary Information). A total of 90 specimens of sea urchins *Paracentrotus lividus* (45 specimen per each site), 30 l of seawater (15 per site), 40 samples (20 per site) of sea grass *Posidonia oceanica* (less than 5 cm leaf fragments, according to the institutional and national ethical guidelines) were collected and analyzed together with 30 l of brackish water from site B (15 l from each creek).

The samplings activity was authorized by the Capitaneria di Porto of Palermo with protocol number: 0029430. In the absence of data about PFOA contamination in the most recent report about chemical contamination in the coastal region subjected to this study^31^, the choice of sample sites was based on supposedly different status of pollution based on the site position or proximity to human activities (e.g. restaurants, pipeline, sewages, etc.).

Site A (see Fig. 1d), was chosen assuming a lower state of pollution based on its position in proximity to Capo Zafferano, at the northern extremity of S. Elia’s bay, with an average depth of 11 m and rocky seabed (see Fig. 1b and Supplementary Information: Table S1). Conversely, Site B (see Fig. 1e was chosen in the same coastal area (only 4.7 km away from Site A) assuming a higher state of pollution due to its position located on the southern side of Solanto promontory, nearby a pipeline and the mouths of two small creeks from inland, with a shallow (3 m) sandy seabed and where a bathing prohibition order is in place^32^ (see Fig. 1b, c and Supplementary Information: Table S1).

The biodistribution of PFOA in the various matrices was evaluated by analyzing sea urchin’s coelomocytes (CC) (90 samples) and coelomic fluid (CF) (90 samples), as well as gonads (G) (63 samples from 32 sea urchins collected in site A and 31 sea urchins collected in site B), or mixed organs (MIX) (27 samples from 13 sea urchins collected in site A and 14 sea urchins collected in site B) consisting of a homogenized mixture of urchin’s inner matrices when gonads were not developed enough for sampling. Due to their mutually exclusive nature the latter two datasets (G and MIX) were merged and labelled as “Gonads or Mixed organs” (GoM) for statistical analysis and graphical representations that needed a uniform dataset of 45 items per site. Further details on the collection of matrices and their labelling are described in the Supplementary Information.

The size of the sea urchins (horizontal diameter without spines) ranged between 30 and 51 mm indicating specimen that have lived in their respective site approximately from 3 to 5 years^25^.

### PFOA extraction and analysis

Materials, equipment and software are described in the Supplementary Information.

PFOA extraction procedures were adapted^33^ to the type of matrix to be analyzed. Recovery percentages (R %) were checked per each batch of analyses by spiking blank samples with different amounts of PFOA analytical standard before the extraction procedure^33^.

Spiked samples underwent the same extraction procedure of unspiked samples and the percentage of recovery R was calculated according to Eq. 1, where C_spike_ is the known concentration of spiked PFOA, D_spiked_ is the instrumental (LC–MS) analytical response of the spiked sample (i.e. the “detected” concentration), D_unspiked_ is the analytical response of the unspiked sample. R was then used in Eq. 2 to calculate the actual values, [PFOA], of PFOA concentrations in unspiked analyzed samples.

1 $$ {\text{R}} = 100 \times \left( {{\text{D}}_{{{\text{spiked}}}} - {\text{D}}_{{{\text{unspiked}}}} } \right)/{\text{C}}_{{{\text{spike}}}} $$

2 $$ \left[ {{\text{PFOA}}} \right] = 100 \times {\text{D}}_{{{\text{unspiked}}}} /{\text{R}} $$

With the exception of [PFOA]_seawater_ and [PFOA]_creek_, which are expressed as nanograms per liter (ppt), all other PFOA concentrations are expressed in nanograms per gram of matrix (ppb).

The PFOA standard was used for calibration before each batch of analyses and a linear response (R^2^ > 0.99) was recorded in the concentration range from 0.1 to 1000 ppb. The RSDs on three replicates were below 10%. LOD (0.1 ppb) and LOQ (1.0 ppb) were quantified by IUPAC method. LC–MS analyses were performed in the negative ion-monitoring mode (see Supplementary Information).

For the analysis of *P. lividus* specimens, an estimate of the total PFOA concentration, [PFOA]_TOT_ in ng/g, in each sea urchin has been calculated considering the sampled weight (W) in grams of each matrix (Eq. 3):

3 $$ \left[ {{\text{PFOA}}} \right]_{{{\text{TOT}}}} = \left( {{\text{W}}_{{{\text{CF}}}} \left[ {{\text{PFOA}}} \right]_{{{\text{CF}}}} + {\text{W}}_{{{\text{CC}}}} \left[ {{\text{PFOA}}} \right]_{{{\text{CC}}}} + {\text{W}}_{{{\text{GoM}}}} \left[ {{\text{PFOA}}} \right]_{{{\text{GoM}}}} } \right)/\left( {{\text{W}}_{{{\text{CF}}}} + {\text{W}}_{{{\text{CC}}}} + {\text{W}}_{{{\text{GoM}}}} } \right) $$

#### Water analysis

During each one of the 3 sampling campaigns, 2 samples of seawater (5 l from Site A and 5 l from site B) and 2 samples of brackish water (5 l from each creek mouths in site B) were collected for a total of 6 seawater samples and 6 brackish water samples.

Samples were checked for the presence of PFOA by solid phase extraction (SPE) (see Supplementary Information) followed by LC–MS analysis^34^.

The percentage of recovery, calculated according to Eq. 1, was R = 120%. [PFOA]_seawater_ and [PFOA]_creek_ concentrations (ng/L) were determined from analytical data according to Eq. 2.

#### *Posidonia oceanica* analysis

A total of 40 samples of leaves were collected from different individuals of *P. oceanica* (20 samples from site A and 20 samples from site B). Each sample was cut in tiny pieces and homogenized using an agate mortar and pestle, weighed (0.5 g) and transferred to a glass tube for extraction (see Supplementary Information).

The percentage of recovery, calculated according to Eq. 1, was R = 70%. [PFOA]_*P. oceanica*_ concentrations (ng/g) were determined from analytical data according to Eq. 2.

#### Coelomocytes and coelomic fluid analysis

The coelomic fluid, containing also the coelomocyte population, was taken from all the ninety collected specimens (45 per site) by inserting an ultrathin and sharp needle (32G 0.26 mm × 12 mm) of a 1 mL syringe through the peristomal membrane^35^. All samples were centrifuged at 4 °C and 1500 rpm for 5 min in a 5804R refrigerated centrifuge (Eppendorf, Germany) thus separating the supernatant coelomic fluid (**CF**) from the coelomocytes (**CC**). CF and CC were then weighed and placed in different glass tubes for subsequent PFOA extractions (see Supplementary Information).

The percentage of recovery, calculated according to Eq. 1, was R = 28% for CF and R = 68% for CC. [PFOA]_CF_ and [PFOA]_CC_ concentrations (ng/g) were determined from analytical data according to Eq. 2.

#### Gonads analysis

The extraction of PFOA from 63 samples of gonads (32 from Site A and 31 from Site B) was performed with LC–MS grade methanol following the same procedure used for extraction from CF and CC (5 mL for samples greater than 0.5 g samples; 2.5 mL for samples between 0.1 g and 0.5 g). In case of undetected PFOA (considered as zero-values in graphics and statistical data treatment), analyses were repeated for confirmation on concentrated sample extracts.

Twenty spiked samples were prepared from the most abundant samples of gonads (10 spiked samples per site), by adding 25 µL of an aqueous 1 mg/L stock solution of PFOA to 0.25 g of gonads samples. The percentage of PFOA recovery from gonads, calculated according to Eq. 1, was R = 73%. [PFOA]_G_ concentrations (ng/g) were determined from analytical data according to Eq. 2.

#### Mixed organs analysis

In 27 specimens of sea urchins (13 from Site A and 14 from Site B), the developmental status was not sufficient to collect at least 0.1 g of gonad sample. For these individuals, organs remaining after CF and CC collection, mainly intestine and undeveloped gonads, were mixed together and extracted similarly to the other matrices.

Spiked samples were prepared by adding 25 µL of an aqueous 1 mg/L stock solution of PFOA to 0.25 g of mixed organs (**MIX**) samples. The percentage of PFOA recovery from **MIX**, calculated according to Eq. 1, was R = 20%. [PFOA]_MIX_ concentrations (ng/g) were determined from analytical data according to Eq. 2.

### Statistical analyses and graphical data representation

**Figure 2:**
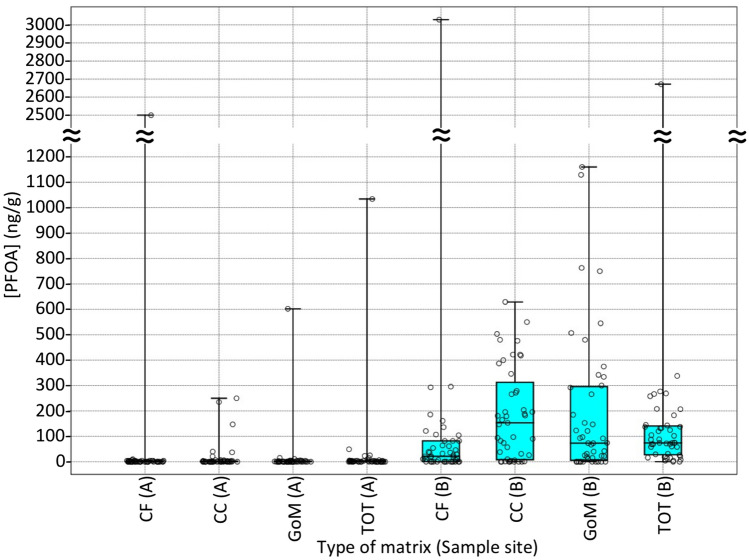
Box and jitter plot showing the concentrations of PFOA found in the Coelomic Fluid (CF) Coelomocytes (CC) and Gonads or Mixed organs (GoM), as well as the total PFOA concentration (TOT), in 45 specimens of *P. lividus* collected from Site A (left side) and in 45 specimens of *P. lividus* collected from Site B (right side).

The distribution of PFOA concentrations in all the sampled matrices from collected sea urchins is graphically represented by box and jitter plot (Fig. 2) where the 25–75 percentiles are drawn using a box; minimum and maximum are shown at the end of the thin lines (whiskers), while the median is marked as a horizontal line in the boxfitting. Statistical tests and linear fittings were used to evaluate data significance and correlations (see Supplementary Information).

A permutational multivariate analysis of variance PERMANOVA^36^ was performed to evaluate the differences in the PFOA concentrations between the two groups of sea urchins collected from site A and site B. The experimental design comprised of one factor (Site) two levels (fixed and orthogonal) and four variables corresponding to the concentrations of PFOA in each type of sample analysed (coelomocytes, coelomic fluid, gonad or mixed organs) including the estimated total PFOA concentration. Each term in the analysis was tested by 999 random permutations.

Finally, Principal Component Analysis (PCA) (see Supplementary Information: PCA tables and graphs) was performed on a dataset, containing five variables. Specifically sea urchin’s size and PFOA concentrations in each type of sample (CF, CC, and GoM) as well as in the entire sea urchin (TOT), to verify the multivariate nature of data in a relatively small number of dimensions, thus limiting the loss of information.

## Results and discussion

### PFOA analyses in water

The concentration of PFOA in seawater from Site A ranged from 1 to 4 ng/L with a median value of [PFOA]_seawater_ = 2 ng/L; these values were about one third of those found in Site B (median [PFOA]_seawater_ = 6 ng/L with an interval of 3–13 ng/L) thus supporting the hypothesized difference of the two sites in terms of chemical contamination (Welch's t-test: α = 0.05; *p*-value < 0.001). Moreover, the median [PFOA]_creek_ = 16 ng/L with a concentration range of 5–34 ng/L suggested that the stream is one of the possible sources of PFOA pollution in Site B.

### PFOA analyses in *P. Oceanica*

The different PFOA contamination of the two sites was also confirmed by the analysis of PFOA in *P. oceanica* (Supplementary Information: Table S2) showing a 45% frequency of contaminated samples from site B, with an average [PFOA]_*P. oceanica*_ = 67 ng/g, while only one sample from Site A was contaminated and with a much lower content of PFOA (13 ng/g) (Welch's t-test: α = 0.05; *p*-value = 0.006). Interestingly, PFOA contamination levels in *P. oceanica* were three orders of magnitude higher than [PFOA]_seawater_ from the corresponding site, thus suggesting the bioaccumulation capacity of *P. oceanica* (see Table 1).

**Table 1:** Bioconcentration factors (BCF) calculated for each analyzed matrix collected from Site B specimens.

Matrix—Species	log_10_BCF
***P. oceanica* seagrass**	
Leaves	4.05
***P. lividus* sea urchin**	
Coelomic fluid	4.28
Coelomocytes	4.48
Gonads	4.50
Mixed organs	4.51
Entire individual	4.41

### PFOA analyses in *P. lividus*

Concerning the whole population of *P. lividus* specimens, the presence of PFOA was detected in 96% of individuals collected from Site B and only in 47% of *P. lividus* specimens from Site A. The detection of PFOA in specimens from site A should be justified also by the presence of PFOA in the seawater from the same site, although at a threefold lower concentration than that found in Site B. Moreover, Welch's t-test confirmed that the distribution of the total concentration of PFOA (log values) in each individual collected from Site B was significantly different (*p* < 0.001) from that recorded in specimens from Site A.

Median values of PFOA concentrations were 0 (for all matrices) for specimens collected from Site A, while they were 21 (in CF), 153 (in CC), and 73 ng/g (in GoM) for specimens collected from Site B (Fig. 2).

These PFOA concentrations were at least three orders of magnitude higher than those recorded in the seawater from the sampling sites suggesting a high PFOA bioaccumulation capacity of *P. lividus* (probably also for its longevity*)* and the opportunity to use this species as bioindicator of PFOA-contamination*.*

Considering the wide range of recorded concentrations, the average level of PFOA contamination in the various matrices was instead evaluated by calculating the median values of PFOA concentration followed by the range of concentration levels.

Such [PFOA] median values in specimens collected from the less contaminated Site A were 0 for all matrices with a wide concentration range of 0–2500 ng/g in CF, 0–250 ng/g in CC, 0–602 ng/g in G, 0–1034 ng/g in TOT due to an extremely limited number of contaminated specimens. Since the home range of *P. lividus* is limited to a few meters per month^37^, our hypothesis is that high concentrations found sporadically in specimens from site A could be due to episodically contamination (e.g. from grazing of PFOA-containing debris).

On the other hand, in specimens collected from Site B, [PFOA] median values were 21 ng/g in CF (range: 0–3029 ng/g), 153 ng/g in CC (range: 0–629 ng/g), 195 ng/g in G (range: 0–1160 ng/g), and 74 ng/g in TOT (range: 0–2672 ng/g). In this regard, the bioaccumulative potential of PFOA was evaluated by calculating the bio-concentration factor (BCF) according to Eq. 4^38,39^.

4 $$ {\text{BCF}}\;\left( {{\text{L}}/{\text{kg}}} \right) = \left[ {{\text{PFOA}}} \right]_{{{\text{MATRIX}}}} \;\left( {{\text{mg}}/{\text{kg}}} \right)/\left[ {{\text{PFOA}}} \right]_{{{\text{seawater}}}} \;\left( {{\text{mg}}/{\text{L}}} \right) $$

The results (log BCF) obtained were used to assess the bioaccumulation behavior of PFOA in *P. lividus* as showed in Table 1.

Considering the European Union criterion of bioaccumulability^38^, which defines a log_10_BCF threshold of 3.7, data reported in Table 1 confirm the very bioaccumulative nature of PFOA in *P. lividus* matrices.

**Figure 3:**
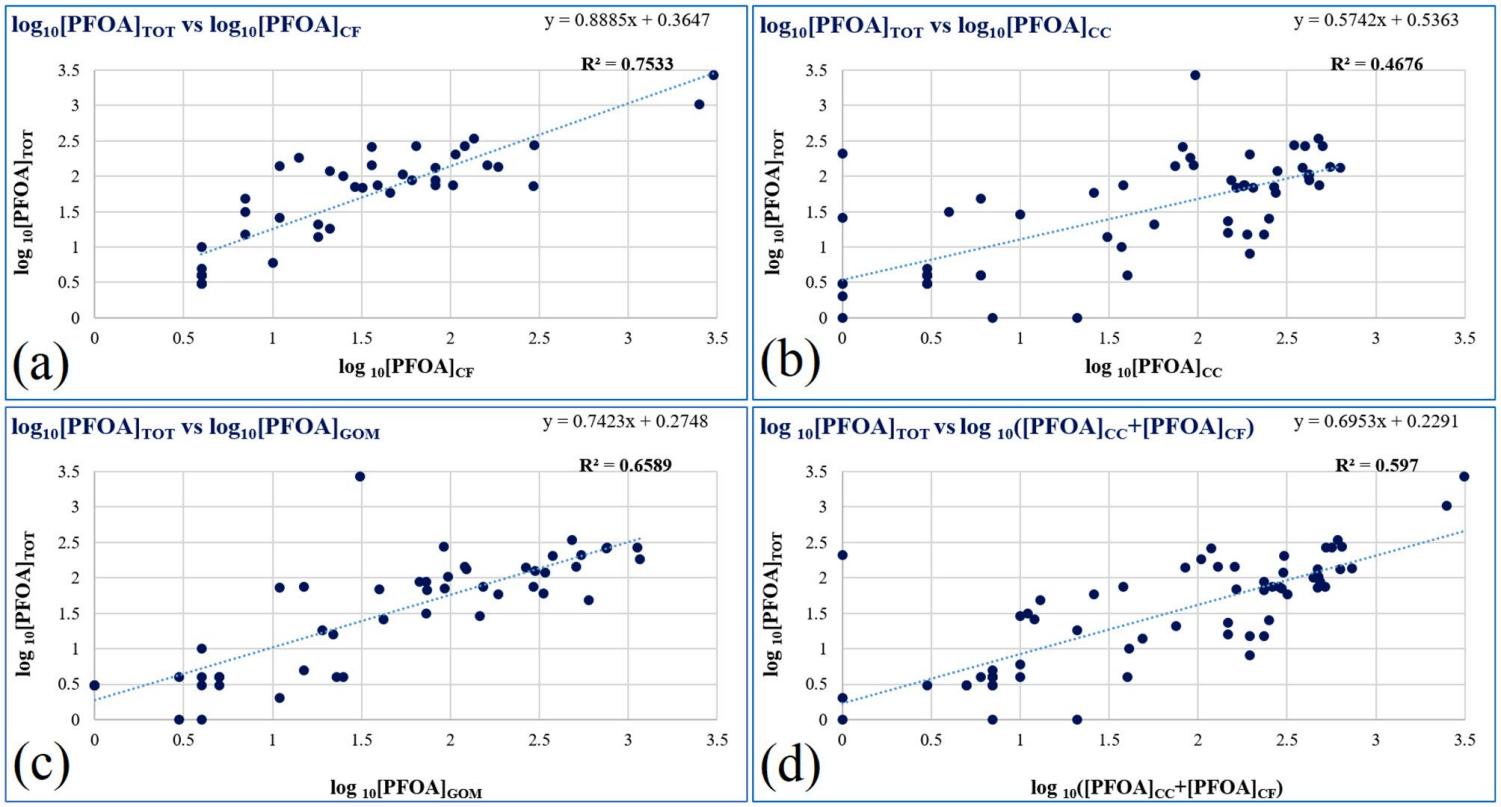
Correlation graphs between either log_10_[PFOA] in a type of matrix labelled as: (**a**) CF = Coelomic Fluid, (**b**) CC = Coelomocytes, (**c**) GoM = Gonads or Mixed organs (x-axis), (**d**) CC + CF versus the total concentration in the entire specimen expressed as log_10_[PFOA]_TOT_ (y-axis).

To further investigate such opportunity for non-invasive PFOA monitoring, we have performed a statistical analysis to evaluate the correlation between the log-trasformed PFOA concentrations in a given matrix log_10_[PFOA]_MATRIX_ versus the total concentration log_10_[PFOA]_TOT_ in the specimen (*i.e.* to evaluate how much a given matrix could be representative of the PFOA contamination in the entire specimen). Of all the combinations (Fig. 3) made in the entire dataset of 90 *P. lividus* specimens, excluding data points corresponding to [PFOA] = 0, log_10_[PFOA]_CF_ showed the best correlation (R^2^ = 0.7533) with log_10_[PFOA]_TOT_ (Fig. 3a), while the correlation between log_10_[PFOA]_TOT_ and log_10_[PFOA] in the other matrices (CC or GoM) was lower (Fig. 3b, c).

The above analysis suggested that log_10_[PFOA]_CF_ could be used to evaluate PFOA contamination in *P. lividus,* also validated by F-test with a significance level of 0.05 and a *p*-value of 0.879. However, by looking at the dataset (see Supplementary Information: Table S3), there are a few cases where PFOA was not detected in CF of PFOA-contaminated sea urchins; thus, the analysis of CF-only would have produced 23.3% of false negative responses. Reasonably, since the sampling of CF intrinsically allows also for CC sampling, the more reliable choice for non-invasive PFOA monitoring would be to analyze both CF and CC matrices. In fact, if we consider the sum of [PFOA]_CF_ and [PFOA]_CC_, subsequently transformed into logarithmic values (Fig. 3d), the risk of false negative responses drops down to 3.3%.

### PCA and PERMANOVA analysis

The dataset (Supplementary Information: Table S3) containing the values of five variables per each sea urchin—specifically, size, [PFOA]_CF_, [PFOA]_CC_, [PFOA]_GoM_, and [PFOA]_TOT_,—was also subjected to principal components analysis (PCA) that generated five principal components (F1–F5) among which the first three represent the system with an 89% of cumulative variability (see Supplementary Information: PCA tables and graphs).

**Figure 4:**
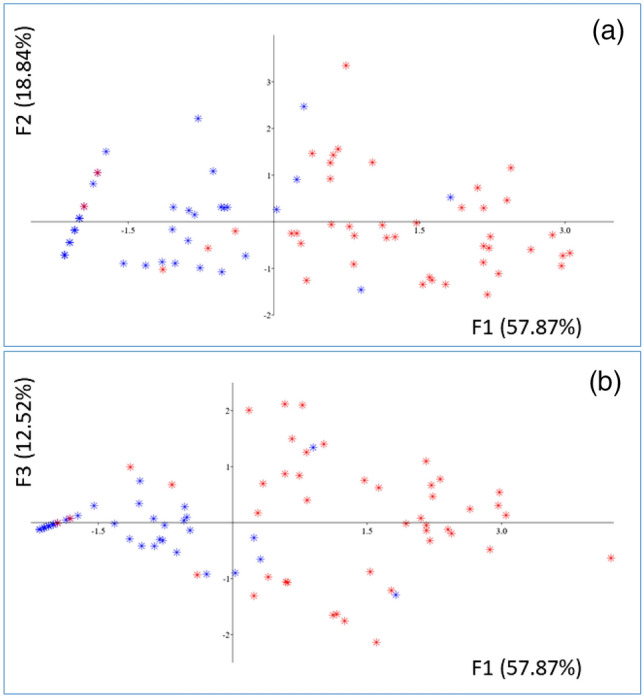
Data distribution by (**a**) 2D-scattered plot of the first two principal components (eigenvectors F1–F2); (**b**) 2D-scattered plot of first and third principal components (eigenvectors F1–F3). Data points arising from specimen collected in Site A (blue symbols) or Site B (red symbols).

In 2D-plotting the scattered distribution of the F1–F2 eigenvectors (principal components) values (Fig. 4a), and distribution of the F1–F3 eigenvectors (Fig. 4b), it is possible to observe separation between points arising from data regarding specimens collected in less contaminated Site A (blue symbols in Fig. 4) and those arising from specimens collected in Site B (red points in Fig. 4), which contained a higher level of PFOA.

However, while specimens from the less contaminated site are mostly distributed in the negative F1 region (see Fig. 4 blue points), specimens from site B are more scattered and, with few exceptions, occupy the positive F1 region (see Fig. 4 red point). In fact, in the wild environment, possible phenomena that may differentiate the level of contaminations within specimens collected from the same site could be: grazing of PFOA-contaminated sediments, spawning, different exposure to currents, etc.

The PCA seems to show a separation between the two sample sites, although without a clear clusterization. Conversely, the PERMANOVA analysis, showed a significant difference between the two sites (*p* = 0.001).

## Conclusions

This study represents the first assessment of PFOA levels in different matrices of adult sea urchins from wild environment demonstrating both PFOA uptake and bioaccumulation in *P. lividus* specimens. Analyses on seagrass *P. oceanica* highlighted the presence of PFOA in the trophic network, with an elevated rate of bioconcentration compared to PFOA levels in the seawater from the collection site. Biodistribution data in the different compartments of *P. lividus* showed that coelomocytes and gonads accumulated the highest levels of PFOA. Bioaccumulation data in *P. lividus* (log_10_BCF > 3.7) confirmed the very bioaccumulative nature of PFOA.

PFOA content of coelomic fluid (CF) and coelomocytes (CC) correlated with the total amount of PFOA in the sea urchin suggesting non-destructive sampling of these matrices for periodical biomonitoring purposes. Additionally, PERMANOVA and Welch’s test analyses demonstrated that the level of PFOA contamination was significantly different between the two sample sites highlighting the opportunity to employ *P. lividus* as a bioindicator of PFOA contamination in the marine environment.

## Supplementary Information

Supplementary Information 1.
