# Supplementary material for: Presence and biodistribution of perfluorooctanoic acid (PFOA) in Paracentrotus lividus highlight its potential application for environmental biomonitoring
Source: Sci Rep. 2021 Sep 21;11:18763. doi: 10.1038/s41598-021-98284-2 (PMC8455602; doi:10.1038/s41598-021-98284-2)
Supplement: Supplementary file 1 — Supplementary Information 1. [file 41598_2021_98284_MOESM1_ESM.pdf]

Presence and biodistribution of Perfluorooctanoic Acid (PFOA) in *Paracentrotus lividus* highlight potential application for environmental biomonitoring.

Dario Savoca[a], Raffaella Melfi[a], Antonio Palumbo Piccionello[a], Salvatore Barreca[b], Silvestre Buscemi[a], Vincenzo Arizza[a], Marco Arculeo[a], Andrea Pace[a]*

[a]*Dipartimento di Scienze e Tecnologie Biologiche, Chimiche e Farmaceutiche (STEBICEF), Università degli Studi di Palermo, 90100 Palermo, Italy*
[b]*Department of Pharmaceutical Sciences, Università degli Studi di Milano, Via L. Mangiagalli 25, 20131 Milan, Italy*

*Corresponding author

E-mail address: andrea.pace@unipa.it ; Phone: +39 091 238 97543

Matrices collection________________ pag.: S2

Materials, equipment and software________________ pag.: S3

PFOA Analyses________________ pag.: S3

Solid phase extraction of water samples________________ pag.:S4

***P. oceanica* analysis**________________ pag.:S4

Coelomocytes and coelomic fluid extraction and analysis________________ pag.:S4

Statistical Analyses and graphical data representation________________ pag.:S5

Table S1: Sampling Campaigns________________ pag.: S6

Table S2: PFOA presence in *P. oceanica*________________ pag.: S7

Figure S1: Schematics of sampled compartment/matrices in *P. lividus*________________ pag.: S7

Table S3: Dataset of PFOA levels in *P. lividus*________________ pag.: S8

Correlation significance________________ pag.: S10

PCA tables and graphs________________ pag.: S11

References________________ pag.: S16

Matrices collection

Different matrices have been sampled from each one of the 90 specimens of *P. lividus* (45 per Site) (see Supplementary Information: Fig. S1) and labelled as follows: 90 samples of coelomocytes (**CC**) and 90 samples of coelomic fluid (**CF**) were collected separately from all the specimens; 63 samples of gonads (**G**) were obtained from 32 sea urchins collected in site A and 31 sea urchins collected in site B; for the remaining 27 specimens, where the status of development of individual organs did not allow to obtain a sample of at least 100 mg, matrices were mixed together and labelled as "mixed organs" (**MIX**). When collected separately, intestine/feces (39 samples from Site A and 28 from Site B) showed an extremely low percentage of recovery and were excluded from the study. The sex of each specimen was determined only when isolated gonads were sexually mature for spawning. Thus, the collected population of *P. lividus* consisted of 27 males (19 from Site A, 8 from Site B), 16 females (6 from Site A, 10 from Site B) and 47 specimens (20 from Site A, 27 from Site B) of undetermined sex. Each sample has been analyzed individually. For the sake of clarity it is noteworthy that, for a given specimen of sea urchin, the determination of PFOA concentration in mixed organs ($[PFOA]_{MIX}$) is mutually exclusive with respect to the concentration of PFOA in gonads ($[PFOA]_{G}$). Nevertheless, as mentioned above, when statistical analysis or graphical representation suggested the use of the same number of items for the different datasets, data referring to mixed organs (**MIX**) or gonads (**G**) were merged and labelled as “Gonads or Mixed organs” (**GoM** or $[PFOA]_{GoM}$).

P. lividus specimens were brought to the laboratory in a refrigerated ice box immediately after capture and matrix sampling was carried out within the next hour. Isolated matrices were either extracted immediately after their collection or stored at -20 ° C and extracted within 72 hours of their collection.

Materials, equipment and software

LC-MS grade methanol (from Honeywell) was used for extractions (including SPE cartridges preconditioning) and analyses. LC-MS grade water (from PanReac Applichem) was used for HPLC-MS analyses and SPE cartridges preconditioning or washing. Ammonium acetate (from Aldrich) was used as additive for HPLC eluents. Perfluorooctanoic acid analytical standard (>98% from Aldrich) was used for LC-MS calibration curves and to obtain spiking solutions which were freshly prepared and checked for their PFOA content before each batch of analyses. SPE cartridges Strata TM-X-AW (33 µm polymeric weak anion 200 mg/6 mL tubes) were purchased from Phenomenex and used for PFOA extraction from seawater samples. PFOA-free polypropylene micropipette tips were used for quantitative small volume withdrawals. In order to prevent any type of PFOA contamination, the equipment used for sampling and extraction procedures was washed with methanol from the same batch of that used for extraction and analysis. LC-MS analyses were performed using a 6540 UHD Accurate-Mass Q-TOF LC/MS (Agilent Technologies) equipped with a Dual AJS ESI source. Box and jitter graphs have been created using PAST software 3.25, as well as F-test and principal component analysis (PCA)[1]. The Welch's t-test, scatter plots and linear correlations have been realized using Excel 2016 (Microsoft).

PFOA Analyses

Analyses were run in triplicate and were performed on an Infinity 1260 HPLC connected to an ESI-QTOF UHD 6540 MS detector operating in the negative ion-monitoring mode. A volume of 10 µL of sample from extracts or standard solutions was injected in a Poroshell EC-C18 3.0 × 50 mm 2.7 µm column and eluted using a mixture of water, containing 4 mM ammonium acetate, and methanol as eluents with a fixed flow of 0.4 mL/min. The following gradient was used for eluition: from Water/MeOH 50/50 (vol/vol) to Water/MeOH 5/95 in 2 min, and then Water/MeOH 2/98 in 1 min and maintaining elution with Water/MeOH 2/98 for further 4 min before returning to Water/MeOH 2/98 in 1 min, then Water/MeOH 95/5 in 2 min and maintaining elution with Water/MeOH 95/5 for further 5 min before returning to initial conditions during an interval time of 3 min. Data were analyzed using the Agilent MassHunter Workstation. The PFOA was identified through MS spectrum (monitoring [M-H]- ion at 412.9664 ± 0.0005 Dalton) and targeted MS/MS (monitoring the 412.966 ± 0.002 Dalton => 368.976 ± 0.002 Dalton fragmentation), and showed a retention time of 4.5 min. PFOA was not detected in blank (MeOH) analyses carried out every two runs, thus confirming the absence of cross contamination. Quality checks were performed by analyzing a 10 ppb PFOA standard solution every ten samples.

Solid phase extraction of water samples

Briefly, the Strata TM-X-AW SPE cartridges were preconditioned by eluting with 5 mL of methanol followed by 5 mL of water. Then, 500 mL of either seawater or creek's water were passed through the cartridge at a rate of 2 drops per second. The cartridge was then washed with 6 mL of water and allowed to dry. The fraction containing the target analyte was eluted with 10 mL (2 x 5 mL) of methanol and reduced to 1 mL under vacuum.

Spiked samples were prepared by adding 20 µL of an aqueous 1 mg/L stock solution of PFOA to 500 mL of either seawater or creek's water.

P. oceanica analysis

After addition of methanol (5 mL) each *P. oceanica* sample was sonicated for 20 min at room temperature, centrifuged for 15 min at 3500 rpm, and then the supernatant (1 mL) was taken for HPLC analysis. Spiked samples were prepared by adding 50 µL of an aqueous 1 mg/L stock solution of PFOA to homogenized samples, of 0.5 g each, of *P. oceanica*.

Coelomocytes and coelomic fluid extraction and analysis

The extraction of PFOA was performed by adding LC-MS grade methanol (5 mL for for samples greater than 0.5 g; 2.5 mL for samples between 0.1 g and 0.5 g) to the sample in a glass vial and sonicating the resulting mixture for 20 min at room temperature. The extraction mixture was then centrifuged for 15 min at 3500 rpm. Then, a portion of the extract supernatant (1 mL) was added to a glass vial to be analyzed by LC-MS. Analyses were run in triplicate and, in case of undetected PFOA, were repeated on a concentrated sample prepared by drying 1 mL of the extract supernatant under a nitrogen current and redissolving the residue in 100 µL of LC-MS grade methanol. Confirmed undetected PFOA were considered as zero-values in both graphs and statistical analyses.

Spiked samples were prepared by adding 50 µL of an aqueous 1 mg/L stock solution of PFOA to 0.5 g samples of either CF or CC.

Statistical Analyses and graphical data representation

Welch's t-test was used to highlight significant differences between $\log_{10}$[PFOA]seawater values recorded from the two sampling sites (site A and B). Moreover Welch's t-test was used also to check whether the mean values of $\log_{10}$[PFOA]TOT from the two populations of *P. lividus* collected from either Site A (specimens 1-45) or Site B (specimens 46-90) can be considered as equal (null hypothesis) or significantly different (alternative hypothesis); this test does not assume that the two populations have equal variance. Before the transformation of $[PFOA]_{TOT}$ into $\log_{10}[PFOA]_{TOT}$, all $[PFOA]_{TOT}$ values were increased by 1 ppb unit in order to avoid the occurrence of indefinite values of log10$[PFOA]_{TOT}$ for concentrations of $[PFOA]_{TOT}$ equal to 0.

Scatter plots and linear fittings were used to verify the existence of correlations between the $\log_{10}$[PFOA] in a given matrix of a *P. lividus* specimen and $\log_{10}[PFOA]_{TOT}$ in the same individual for the entire group of 90 collected sea urchins. F-test of overall significance were performed to verify if such linear regression models provide a better fit to the data than a model that contains no independent variables.

Sampling campaigns

Three sampling campaigns were carried out at the two sample sites chosen for this study. The main features of the sites and sampling details are summarized in Table S1 below.

Table S1

Sampling Site	Geographical Coordinates	Seabed	Max Depth (m)	Water streams (creeks) from inland	Bathing restrictions due to pollution
Site A	38°03’54’’N 13°32’22’’E	Mostly rocky	11	NO	NO
Site B	38°06’27’’N 13°32’19’’E	Mostly sandy	3	YES	YES

Sampling Campaign	Sampling Period	Sample Site	Collected Samples				
			Seawater (liters)	Creek 1 (liters)	Creek 2 (liters)	seagrass: *Posidonia oceanica* (# of samples)	sea urchins: *Paracentrotus lividus* (# of specimens)
Campaign I	October 2018	A	5	-	-	10	15
Campaign I	October 2018	B	5	5	5	10	15
Campaign II	March 2019	A	5	-	-	5	15
Campaign II	March 2019	B	5	5	5	5	15
Campaign III	July 2019	A	5	-	-	5	15
Campaign III	July 2019	B	5	5	5	5	15

PFOA presence in *Posidonia oceanica*

Table S2. PFOA concentration in seawater and *P. oceanica* from the two sampling sites.

P.oceanica **Sample #**	**Sampling Site**	[PFOA]$_{P.\ oceanica}$ **(ng/g)**	**Sampling Site**	[PFOA]$_{P.\ oceanica}$ **(ng/g)**
1	A	0	B	13
2	A	0	B	7
3	A	0	B	6
4	A	0	B	294
5	A	0	B	41
6	A	0	B	71
7	A	0	B	6
8	A	0	B	794
9	A	13	B	0
10	A	0	B	0
11	A	0	B	0
12	A	0	B	0
13	A	0	B	0
14	A	0	B	0
15	A	0	B	0
16	A	0	B	0
17	A	0	B	0
18	A	0	B	107
19	A	0	B	0
20	A	0	B	0

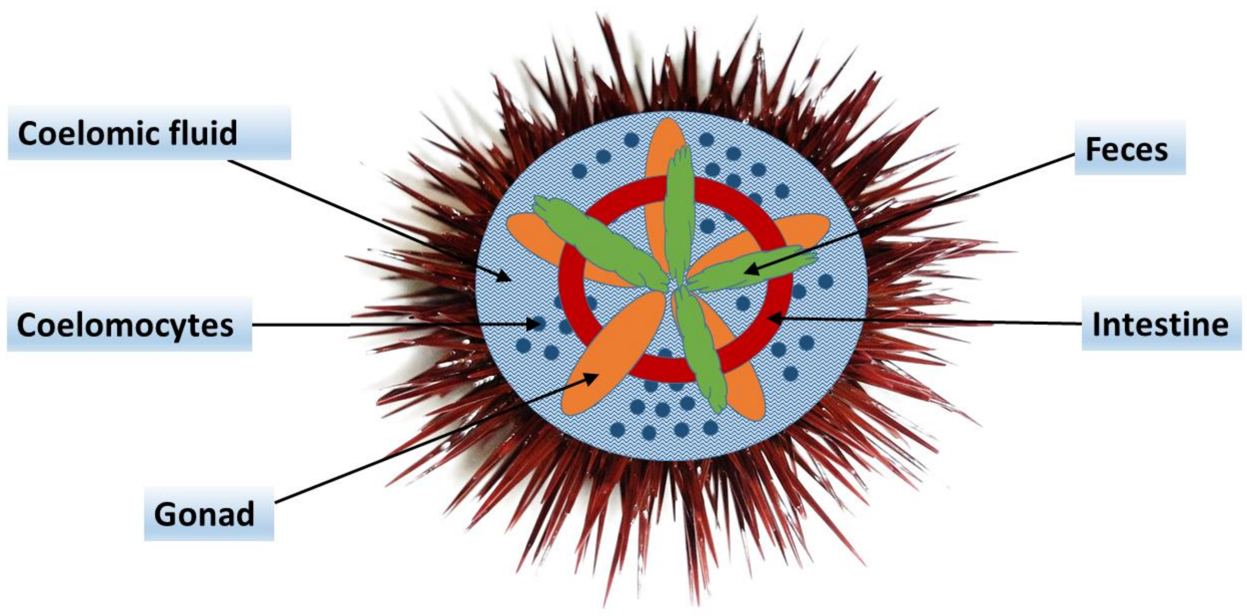

Figure S1. Schematics of sampled compartment/matrices in *P. lividus*

Dataset and correlation

Table S3. Dataset of PFOA levels in *Paracentrotus lividus*

P.lividus Specimen #	Sex	Size (cm)	$[PFOA]_{CF}$ (ng/g)	$[PFOA]_{CC}$ (ng/g)	$[PFOA]_{Mix}$ (ng/g)	$[PFOA]_{G}$ (ng/g)	$[PFOA]_{GoM}$ (ng/g)	$[PFOA]_{Water}$ (ng/l)	Sampling Site	$[PFOA]_{TOT}$ (ng/g)
1	Male	3.5	4	6		3	3	2	A	4
2	Male	3.5	4	3		5	5	2	A	4
3	Male	3	4	3		1	1	2	A	3
4	Undetermined	3	7	6		602	602	2	A	49
5	Male	3.5	0	1		11	11	2	A	2
6	Male	3.5	4	3		4	4	2	A	4
7	Male	3.8	4	37		4	4	2	A	10
8	Male	3	4	3		15	15	2	A	5
9	Male	3.3	0	3	5		5	2	A	3
10	Male	3	0	7		3	3	2	A	1
11	Female	3.2	4	1		4	4	2	A	3
12	Undetermined	3	4	1		1	1	2	A	3
13	Undetermined	3	0	1		4	4	2	A	1
14	Undetermined	3.4	4	3		1	1	2	A	3
15	Undetermined	3	4	3	5		5	2	A	4
16	Undetermined	3	0	0		0	0	2	A	0
17	Undetermined	3.1	0	0		0	0	2	A	0
18	Undetermined	3.2	0	0		0	0	2	A	0
19	Undetermined	3.4	0	0		0	0	2	A	0
20	Undetermined	3.6	0	0		0	0	2	A	0
21	Undetermined	3.1	0	0		0	0	2	A	0
22	Undetermined	3.2	0	0		0	0	2	A	0
23	Undetermined	3.2	0	0		0	0	2	A	0
24	Male	3.3	0	0		0	0	2	A	0
25	Female	4.5	0	235		0	0	2	A	15
26	Female	4.3	0	21		0	0	2	A	1
27	Male	3.3	0	0	0		0	2	A	0
28	Female	3.3	0	0		0	0	2	A	0
29	Male	3.7	0	0		0	0	2	A	0
30	Male	3.5	0	250		0	0	2	A	25
31	Male	3.6	2500	0	0		0	2	A	1034
32	Male	3.4	10	0		0	0	2	A	6
33	Female	3.8	0	40	0		0	2	A	4
34	Male	3.9	0	0	0		0	2	A	0
35	Male	3.1	0	147	0		0	2	A	23
36	Undetermined	3.3	0	0		0	0	2	A	0
37	Undetermined	3.3	0	0	0		0	2	A	0
38	Undetermined	3.7	0	0	0		0	2	A	0

P.lividus **Specimen #**	**Sex**	**Size (cm)**	**[PFOA]$_{CF}$ (ng/g)**	**[PFOA]$_{CC}$ (ng/g)**	**[PFOA]$_{Mix}$ (ng/g)**	**[PFOA]$_{G}$ (ng/g)**	**[PFOA]$_{GoM}$ (ng/g)**	**[PFOA]$_{Water}$ (ng/l)**	**Sampling Site**	**[PFOA]$_{TOT}$ (ng/g)**
39	Male	3.2	0	0	0		0	2	A	0
40	Undetermined	3.1	0	0	0		0	2	A	0
41	Undetermined	3	0	0	0		0	2	A	0
42	Undetermined	3	0	0	0		0	2	A	0
43	Undetermined	3.1	0	0		0	0	2	A	0
44	Male	3.4	0	0		0	0	2	A	0
45	Female	3	0	0		0	0	2	A	0
46	Undetermined	3.4	0	165		40	40	6	B	69
47	Undetermined	3.5	296	346		92	92	6	B	277
48	Undetermined	3.3	7	188	0		0	6	B	15
49	Undetermined	3.3	0	196	0		0	6	B	8
50	Undetermined	3.4	46	272		0	0	6	B	58
51	Undetermined	3.4	0	629	0		0	6	B	132
52	Undetermined	3.3	104	182	0		0	6	B	75
53	Undetermined	3.3	25	418	0		0	6	B	102
54	Undetermined	3.4	39	480		153	153	6	B	75
55	Male	4	18	31		0	0	6	B	14
56	Undetermined	3.4	0	0		0	0	6	B	0
57	Undetermined	5.1	3029	97		31	31	6	B	2672
58	Male	5	21	0		19	19	6	B	18
59	Female	4	18	57		0	0	6	B	21
60	Undetermined	3.2	0	6		23	23	6	B	4
61	Undetermined	3.5	161	0		121	121	6	B	143
62	Male	4.2	0	10		147	147	6	B	29
63	Female	4	11	1		42	42	6	B	26
64	Female	3.4	0	0		334	334	6	B	60
65	Undetermined	3.2	0	38		292	292	6	B	74
66	Undetermined	3.4	107	196	375		375	6	B	206
67	Undetermined	3	82	387		123	123	6	B	132
68	Undetermined	3	82	181	15		15	6	B	74
69	Undetermined	3.2	36	82	750		750	6	B	258
70	Female	3.1	11	74		266	266	6	B	138
71	Undetermined	3	7	4		73	73	6	B	31
72	Undetermined	3.4	136	476	480		480	6	B	338
73	Undetermined	3.5	0	1	545		545	6	B	208
74	Undetermined	3	0	0		25	25	6	B	4
75	Female	3	293	179		11	11	6	B	72
76	Male	3.7	0	0	0		0	6	B	0
77	Undetermined	4.1	0	26	185		185	6	B	58
78	Undetermined	3.9	0	0	300		300	6	B	125
79	Male	3.7	32	204		74	74	6	B	68
80	Male	4.1	61	422		67	67	6	B	87
81	Female	3.9	29	266		93	93	6	B	70

P.lividus Specimen #	Sex	Size (cm)	$[PFOA]_{CF}$ (ng/g)	$[PFOA]_{CC}$ (ng/g)	$[PFOA]_{Mix}$ (ng/g)	$[PFOA]_{G}$ (ng/g)	$[PFOA]_{GoM}$ (ng/g)	$[PFOA]_{Water}$ (ng/l)	Sampling Site	$[PFOA]_{TOT}$ (ng/g)
82	Undetermined	3.7	82	153	73		73	6	B	88
83	Female	3.8	54	422		97	97	6	B	105
84	Female	3	186	550		0	0	6	B	137
85	Undetermined	3.5	21	279		342	342	6	B	120
86	Undetermined	3.4	64	503		1129	1129	6	B	267
87	Female	3.3	121	400		763	763	6	B	269
88	Female	3.3	14	90		1160	1160	6	B	183
89	Male	3.5	0	147		22	22	6	B	16
90	Male	3.4	36	94		507	507	6	B	145

Correlation significance

The correlation of $\log_{10}[PFOA]_{TOT}$ vs $\log_{10}[PFOA]_{CF}$ (Fig. 3 a) had R^2= 0.75 (thus a correlation coefficient R = 0.86) and was performed on 44 data points (we excluded values where [PFOA] =0 because of its undeterminable log). When the number of observables is equal to 44, the probability to obtain R > 0.7 from two non-correlating variables is p << 0.05%[2]. Therefore, observed correlation is significant.
The correlation of $\log_{10}[PFOA]_{TOT}$ vs $\log_{10}[PFOA]_{CC}$ (Fig. 3 b) had R^2= 0.46 (thus a correlation coefficient R = 0.68) and was performed on 58 data points (we excluded values where [PFOA] =0 because of its undeterminable log). When the number of observables is equal to 58, the probability to obtain R > 0.45 from two non-correlating variables is p << 0.05%[2]. Therefore, observed correlation is significant.
The correlation of $\log_{10}[PFOA]_{TOT}$ vs $\log_{10}[PFOA]_{GOM}$ (Fig. 3 c) had R^2= 0.66 (thus a correlation coefficient R = 0.81) and was performed on 49 data points (we excluded values where [PFOA] =0 because of its undeterminable log). When the number of observables is equal to 49, the probability to obtain R > 0.45 from two non-correlating variables is p << 0.05%[2]. Therefore, observed correlation is significant.
The correlation of $\log_{10}[PFOA]_{TOT}$ vs $\log_{10}([PFOA]_{CC}+[PFOA]_{CF})$ (Fig. 3 d) had R^2= 0.59 (thus a correlation coefficient R = 0.77) and was performed on 62 data points (we excluded values where [PFOA] =0 because of its undeterminable log). When the number of observables is equal to 62, the probability to obtain R > 0.45 from two non-correlating variables is p << 0.05%. Therefore, observed correlation is significant.

PCA tables and graphs - Matrix : Correlation

Summary

Factors (F)	Eigenvalue	% variance
1	2.89326	57.865
2	0.942043	18.841
3	0.625781	12.516
4	0.44039	8.8078
5	0.0985259	1.9705

Factor loadings

	F1	F2	F3	F4	F5
Size	0.18696	0.97175	0.10799	0.056867	0.076485
CF	0.48325	-0.036288	-0.38963	-0.70029	0.35061
CC	0.47114	-0.10531	-0.43496	0.70933	0.27312
GoM	0.43382	-0.20271	0.80341	0.038971	0.35175
TOT	0.56687	-0.046892	0.043214	-0.041126	-0.82031

Scores

Observations	F1	F2	F3	F4	F5
1	-0.47576	0.30952	-0.13722	-0.11856	0.1953
2	-0.51129	0.29973	0.097432	-0.27503	0.19151
3	-1.0155	-0.89155	-0.41524	-0.36382	0.01614
4	0.89839	-1.4581	1.3437	-0.31726	0.017777
5	-1.0343	0.31117	0.7455	0.09109	0.13721
6	-0.54399	0.31501	0.036861	-0.27796	0.16499
7	0.23693	0.90622	-0.27082	0.40388	0.16978
8	-0.53473	-1.0748	0.28381	-0.33813	0.16265

9	-1.0437	-0.16428	0.34199	0.24717	-0.035656
10	-1.3188	-0.93605	-0.018472	0.4096	0.18745
11	-0.88259	-0.40473	0.072279	-0.51972	0.11368
12	-1.1496	-0.86158	-0.29144	-0.56571	-0.061598
13	-1.547	-0.8948	0.30327	0.0094097	0.064427
14	-0.81497	0.15092	-0.29939	-0.30281	0.098192
15	-0.75914	-0.98855	-0.045737	-0.35042	0.09011
16	-2.154	-0.71467	-0.12167	-0.20505	0.019193
17	-2.102	-0.44459	-0.091654	-0.18925	0.04045
18	-2.0513	-0.18102	-0.062363	-0.17382	0.061196
19	-1.9534	0.3278	-0.005816	-0.14405	0.10124
20	-1.8599	0.814	0.048217	-0.1156	0.13951
21	-2.102	-0.44459	-0.091654	-0.18925	0.04045
22	-2.0513	-0.18102	-0.062363	-0.17382	0.061196
23	-2.0513	-0.18102	-0.062363	-0.17382	0.061196
24	-2.0018	0.076349	-0.03376	-0.15876	0.081453
25	0.31012	2.4712	-0.65429	1.5368	-0.16028
26	-0.77958	2.2144	-0.31765	0.86204	0.34153
27	-2.0018	0.076349	-0.03376	-0.15876	0.081453
28	-2.0018	0.076349	-0.03376	-0.15876	0.081453
29	-1.8146	1.0492	0.074358	-0.10183	0.15803
30	0.028806	0.26302	-0.89938	1.4169	-0.51286
31	1.8203	0.52357	-1.2907	-2.9087	-1.1992
32	-0.87385	0.24279	-0.41982	-0.99653	-0.23919
33	-0.62412	1.0835	-0.53072	0.96227	-0.026391
34	-1.7269	1.505	0.12501	-0.075156	0.1939
35	-0.29056	-0.73057	-0.91981	1.205	-0.62146
36	-2.0018	0.076349	-0.03376	-0.15876	0.081453
37	-2.0018	0.076349	-0.03376	-0.15876	0.081453

38	-1.8146	1.0492	0.074358	-0.10183	0.15803
39	-2.0513	-0.18102	-0.062363	-0.17382	0.061196
40	-2.102	-0.44459	-0.091654	-0.18925	0.04045
41	-2.154	-0.71467	-0.12167	-0.20505	0.019193
42	-2.154	-0.71467	-0.12167	-0.20505	0.019193
43	-2.102	-0.44459	-0.091654	-0.18925	0.04045
44	-1.9534	0.3278	-0.005816	-0.14405	0.10124
45	-2.154	-0.71467	-0.12167	-0.20505	0.019193
46	0.83095	-0.29797	0.40095	1.3228	-0.41936
47	2.8697	-0.28331	-0.48004	-0.39649	0.24018
48	0.2369	-0.24791	-1.307	0.60782	-0.043251
49	-0.3957	-0.20043	-0.93287	1.3377	-0.17113
50	1.1185	-0.072237	-1.6531	0.10268	-0.18284
51	0.5934	-0.058466	-1.058	1.6391	-1.0568
52	1.2486	-0.32612	-1.7565	-0.30661	-0.2081
53	1.1623	-0.34404	-1.6344	0.40322	-0.46964
54	2.1612	-0.52169	-0.045291	0.39875	0.4885
55	0.3964	1.4647	-0.97135	-0.10568	0.059414
56	-1.9534	0.3278	-0.005816	-0.14405	0.10124
57	4.2202	3.0975	-0.6322	-1.4202	-0.27024
58	0.7442	3.3499	0.8416	-1.0046	0.19745
59	0.61325	1.4306	-1.0698	0.060154	-0.021196
60	-0.67698	-0.56696	0.67851	0.41314	0.12266
61	1.4698	-0.027968	0.75616	-1.8772	-0.22648
62	0.66248	1.5576	1.4981	0.67626	-0.067333
63	0.58319	1.2645	0.87222	-0.7078	-0.0087289
64	0.18221	-0.24996	2.0091	-0.12962	-0.63421
65	0.82397	-0.91053	1.2579	0.90155	-0.36234
66	2.6479	-0.59743	0.24318	-0.20376	0.30184

67	2.2001	-1.5619	-0.32144	0.012792	0.2598
68	1.5341	-1.3449	-0.87814	-0.22965	0.097392
69	2.3152	-1.1129	0.77796	-0.11441	-0.0029933
70	1.6299	-1.2519	0.62351	0.21867	-0.13775
71	0.33834	-1.2579	0.69779	-0.44047	-0.15345
72	3.05	-0.67135	0.13207	-0.032567	0.28758
73	0.77854	-0.10216	2.0998	0.070781	-0.93973
74	-1.1418	-1.0232	0.99336	-0.18358	-0.12594
75	1.7698	-1.342	-1.2114	-0.66682	0.27991
76	-1.8146	1.0492	0.074358	-0.10183	0.15803
77	1.0161	1.2724	1.4037	0.91602	-0.21024
78	0.58231	0.9203	2.119	-0.076443	-0.83613
79	1.9351	0.30245	-0.017393	0.24821	0.34941
80	2.4421	1.1563	-0.19436	0.29088	0.50047
81	2.0997	0.72895	0.079686	0.38733	0.42056
82	2.1613	0.2941	-0.14001	-0.15371	0.37442
83	2.4015	0.46054	-0.12021	0.29509	0.40947
84	1.6036	-1.1881	-2.1383	-0.2395	-0.27798
85	2.2309	-0.31931	0.46717	0.46265	0.28304
86	2.9766	-0.72703	0.54222	0.25517	0.38152
87	2.9634	-0.94704	0.30616	-0.046579	0.38343
88	2.1581	-0.87264	1.0987	0.24792	0.068821
89	0.28049	-0.46488	0.17278	1.2927	0.0082473
90	2.2167	-0.56457	0.6689	-0.040547	0.21581

2D-scattered plot of the first two principal components (eigenvectors F1-F2)

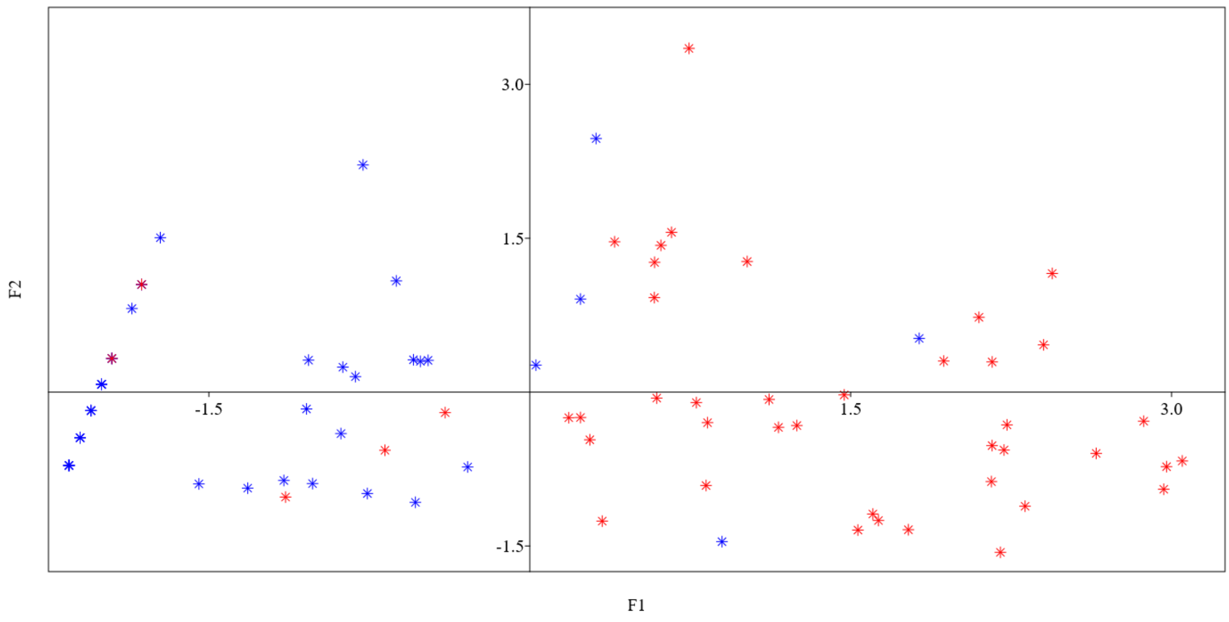

2D-scattered plot of first and third principal components (eigenvectors F1-F3)

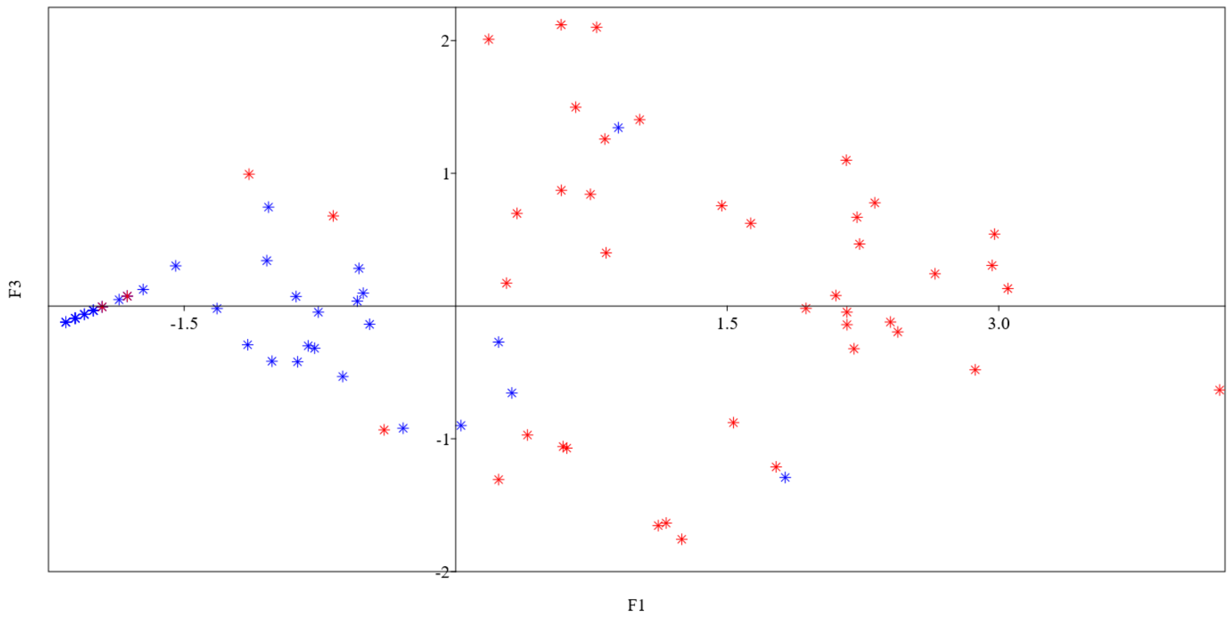

References

1. Hammer, Ø., Harper, D.A.T. & Ryan, P.D. PAST: Paleontological statistics software package for education and data analysis. *Palaeontologia Electronica*, **4**, 1-9. Available online at https://palaeoelectronica.org/2001_1/past/issue1_01.htm.
2. Taylor, J. R. An introduction to error analysis: The study of uncertainties in physical measurements. Mill Valley, Calif: University Science Books (1982).
